# Survival, quality of life, and motor function in brain metastases surgery: The role of complete resection

**DOI:** 10.1093/nop/npaf011

**Published:** 2025-01-31

**Authors:** Rebecca R Winther, Marianne J Hjermstad, Olav Erich Yri, Eva Skovlund, Nina Aass, Guro L Astrup, Stein Kaasa, Cathrine Saxhaug, Einar Osland Vik-Mo

**Affiliations:** Department of Oncology, Oslo University Hospital, Oslo, Norway/European Palliative Care Research Centre (PRC), Department of Oncology, Oslo University Hospital, and Institute of Clinical Medicine, University of Oslo, Oslo, Norway; Department of Oncology, Oslo University Hospital, Oslo, Norway/European Palliative Care Research Centre (PRC), Department of Oncology, Oslo University Hospital, and Institute of Clinical Medicine, University of Oslo, Oslo, Norway; Department of Oncology, Oslo University Hospital, Oslo, Norway/European Palliative Care Research Centre (PRC), Department of Oncology, Oslo University Hospital, and Institute of Clinical Medicine, University of Oslo, Oslo, Norway; Department of Public Health and Nursing, Norwegian University of Science and Technology, NTNU, Trondheim, Norway; Department of Oncology, Oslo University Hospital, Oslo, Norway/European Palliative Care Research Centre (PRC), Department of Oncology, Oslo University Hospital, and Institute of Clinical Medicine, University of Oslo, Oslo, Norway; Department of Oncology, Oslo University Hospital, Oslo, Norway/European Palliative Care Research Centre (PRC), Department of Oncology, Oslo University Hospital, and Institute of Clinical Medicine, University of Oslo, Oslo, Norway; Department of Oncology, Oslo University Hospital, Oslo, Norway/European Palliative Care Research Centre (PRC), Department of Oncology, Oslo University Hospital, and Institute of Clinical Medicine, University of Oslo, Oslo, Norway; Division of Radiology and Nuclear Medicine, University Hospital, Oslo, Norway; Vilhelm Magnus Laboratory, Department of Neurosurgery, Oslo University Hospital, and Institute of Clinical Medicine, University of Oslo, Oslo, Norway

**Keywords:** brain metastases, postoperative motor dysfunction, quality of life, surgery

## Abstract

**Background:**

One in 3 patients with advanced cancer develops brain metastases. Surgical resection of brain metastases is done in 15%–20% of these patients. While gross total resection (GTR) is believed to extend overall survival (OS), concerns exist regarding increased morbidity. This study examines the impact of surgical resection, particularly GTR, on self-reported symptoms, focusing on quality of life (QoL) and motor dysfunction.

**Methods:**

We conducted a prospective cohort study involving adult patients undergoing surgical resection for brain metastases from solid tumors in a defined region of Norway between 2017 and 2021. Clinical data were collected at inclusion prior to surgery and every 3 months the first year. Patients completed monthly questionnaires assessing QoL and motor dysfunction. QoL was measured using the European Organisation for Research and Treatment of Cancer (EORTC) QLQ-C15-PAL, while motor dysfunction was evaluated using the EORTC QLQ-BN20.

**Results:**

A total of 155 patients were included and median OS was 13 months. GTR was achieved in 69 (44%) patients and was associated with longer median OS compared to subtotal resection (17.7 vs. 10.9 months, *P* = .04). Mean QoL remained stable throughout the follow-up period. Improved motor dysfunction 1 month after surgery was reported by 23% of the patients, while 25% reported worse motor dysfunction. Factors associated with a high motor dysfunction score at 1 month were age >70 years, higher baseline motor dysfunction, and multiple brain metastases. Neither GTR nor location of metastases in motor-associated areas were associated with worsened motor dysfunction.

**Conclusion:**

Self-reported QoL is maintained after surgery for brain metastases. Complete resection is associated with extended OS without compromising self-reported motor function.

**Key Points**

1. Quality of life seems to be preserved after surgical resection brain metastases.
2. Complete resection of brain metastases is associated with increased survival and not associated with worse postoperative motor function.

**Importance of the Study**

Brain metastases affect up to one-third of patients with disseminated cancer and cause severe morbidity and mortality. Surgical resection is a key management option. However, there is a lack of knowledge regarding how resection of brain metastases affects quality of life, and how important complete resection is for survival and self-reported symptoms. In this study, we found that patients who underwent surgical resection of brain metastases reported stable quality of life a year after surgery. In addition, complete resection of brain metastases was associated with longer overall survival, without compromising postoperative self-reported motor function, even after surgery in motor areas of the brain. We believe complete resection of brain metastases should be prioritized whenever feasible.

Brain metastases develop in 10%–30% of all patients with cancer from solid tumors, and the incidence is increasing.^1^ The most common primary cancers associated with brain metastases are melanoma, lung, breast, and colorectal cancer.^1–4^ The presence of brain metastases often leads to serious complications, including neurocognitive impairment, headaches, seizures, focal or systemic neurological deficits, and psychological distress,^5^ significantly impacting the quality of life (QoL) of both patients and caregivers.^6,7^ Overall, the median life expectancy after diagnosis of brain metastases is about 6 months, with considerable variation by age and general health, primary tumor, number of brain metastases, available anticancer therapy, and extent of extracranial disease.^8^ In the most favorable prognostic groups, such as patients under 70 years with breast cancer, absence of extracranial disease, and having a single brain metastasis, the median overall survival (OS) can extend to 3 years, with some individuals experiencing continued long-term survival.^9^ However, given that less than 3% become long-term survivors beyond 5 years,^10^ maintaining QoL during and after cancer treatment is paramount.

Patients with brain metastases typically undergo a combination of treatments, including stereotactic radiotherapy, whole-brain radiotherapy, systemic anticancer therapy, and surgical resection.^11^ Surgical intervention aims to alleviate neurological symptoms, prolong survival, and/or obtain a biopsy for diagnosis.^12,13^ Gross total resection (GTR) of brain metastases is associated with improved survival.^14^ Nevertheless, radical tumor removal may pose risks of damaging healthy brain tissue, leading to new neurological deficits. Postoperative assessment by neurosurgeons has revealed new deficits in approximately 26% of patients undergoing surgery for a single brain metastasis.^15^ However, discrepancies often exist between patients’ self-reported symptoms and those recorded by healthcare professionals, highlighting the importance of considering patients’ perspectives.^16–18^ Self-reported motor dysfunction, both pre- and post-surgery, offers valuable insights into patients’ functional status, while self-reported QoL assessments provide valuable information about the impact of brain metastasis surgery on patients’ daily lives.

This study aims to investigate survival, self-reported QoL, and symptoms, including motor dysfunction, in a cohort of patients who underwent surgery as their initial treatment for brain metastases.

Additionally, we sought to address the following research question: Does the extent of resection affect self-reported motor dysfunction following surgery for brain metastases?

## Methods

### Design and Population

The study is part of the study Brain Metastases in Norway (Clinical trials number: NCT03346655), a prospective cohort study investigating treatment patterns and adherence to clinical guidelines in 912 patients diagnosed with brain metastases in the South-East and Middle healthcare regions of Norway. Eligible participants were adults aged ≥18 years with a first-time diagnosis of brain metastases from solid tumors. Exclusion criteria included previous treatment for brain metastases, hematological malignancies, and inability to provide consent due to cognitive impairment, language barriers, or other reasons. Patient self-reported QoL and symptoms were assessed during and after treatment for up to 12 months after inclusion. This substudy focuses on the 155 patients who underwent surgery as their primary treatment for brain metastases between 2017 and 2021. The last date of follow-up was 10.01.2022.

### Brain Metastasis Location, Surgical Techniques, and Extent of Resection

All patients underwent a preoperative MRI shortly prior to surgery. In patients who responded to questionnaires, exact brain metastasis location and relation to areas affecting motor function were reviewed by an experienced neurosurgeon (E.O.V.-M.). The areas considered relevant for motor function were cerebellum, and the primary motor cortex, the supplementary motor area, the premotor cortex, and the corticospinal tract. Surgery was performed at 2 locations within the Department of Neurosurgery, Oslo University Hospital, which is the sole regional provider of brain tumor neurosurgery in South-Eastern Norway. All procedures utilized neuro-navigation, with adjunctive techniques such as intraoperative ultrasound, continuous cortical and subcortical neurophysiological monitoring, and fluorescence visualization using sodium fluorescein or 5-aminolevulinic acid when appropriate. Postoperative evaluation included MRI within 48 h to determine the extent of resection and identify complications, with images reviewed by in-house radiologists.

### Data Collection and Self-Reported Questionnaires

Sociodemographic variables were collected from patients and relevant clinical data from electronic patient records, such as age, sex, primary tumor, extent of disease, comorbidity, and current treatment, at inclusion, supplemented by clinical data on subsequent treatments, complications, and progression collected every 3 months during the first year. To investigate QoL and symptoms, we used self-report questionnaires, the European Organisation for Research and Treatment of Cancer (EORTC) QLQ-C15-PAL,^19^ a questionnaire developed for palliative care patients, and QLQ-BN20,^20^ a questionnaire developed for patients with primary brain tumors and validated for use in brain metastases. In this study, we focused on self-reported motor dysfunction given its significance for level of functioning and activities of daily living plus overall QoL. For motor dysfunction (QLQ-BN20), 3 items make up the scale of motor dysfunction. These involve the patients’ perception of muscle coordination (during the last week, did you have trouble with your coordination?), instability (did you feel unsteady on your feet?), and degree of hemiparesis (did you have weakness on 1 side of the body?). Answers are scored on a 1 (*not at all*) to 4 (*very much*) Likert combined and converted to a 0–100 scale, where higher scores indicate a higher degree of motor dysfunction and 0 is the best outcome, indicating no motor dysfunction. For QoL (QLQ-C15-PAL) patients were asked to indicate their QoL on a scale from 1 (*very poor*) to 7 (*excellent*). The answers are converted into a score from 0 to 100, where higher scores indicate a better QoL.

The first set of questionnaires was completed at the time of inclusion, with most patients being in hospital. The subsequent forms were sent by postal mail every month up to 1 year or until death, whichever came first. Previously identified minimal clinically important differences for QLQ-C15-PAL scores were set at 10 points.^21–23^ Specific thresholds for improvement and deterioration of the motor dysfunction scale have been defined as 5.6 and -−4.4, respectively, in patients with brain metastases.^24^

### Statistical Analyses and Artificial Intelligence (AI) Support

Survival analyses were done with the Kaplan–Meier estimator and log-rank test. Multiple linear regression analyses were performed to assess factors associated with motor dysfunction at 1 month. Model fit was visually assessed with residual plots. Frequencies were compared between groups using chi-square and Fisher’s exact tests. A *P*-value < .05 was considered statistically significant. We performed all statistical analyses in SPSS Statistics 28 (IBM Corp.). ChatGPT was used to improve the language in the manuscript.

### Ethical Approval

We obtained written consent from all patients at the time of inclusion. The Regional Committee for Medical and Health Research Ethics in the South-East and Central health regions of Norway (REK no. 2017/1358), the hospital data protection officer, and the internal review boards approved the study. All data handling and procedures were performed in accordance with GDPR, the 1975 Helsinki declaration and its later amendments.

## Results

### Patient Characteristics and OS

Among the 912 patients enrolled in the Brain Metastases in Norway study, 155 (17%) underwent surgery as their primary treatment for brain metastases and were included in the present study. The median age of the cohort was 66 years (ranging from 21 to 85) and lung, melanoma, breast, and colorectal cancer were the most common primary tumors. A single brain metastasis was present in 109 (70%) patients, and 88 (57%) patients had extracranial metastases at the time of surgery. In total, 105 (68%) patients had an Eastern Cooperative Oncology Group (ECOG) performance status score of 1 (Table 1).

**Table 1.** Patient Characteristics

Patient Characteristics	*N*
Total number of patients	155
Median age (min–max)	66 (21–85) years
Female sex	90 (58%)
Primary cancer	
Lung	51 (33%)
Melanoma	30 (19%)
Breast	25 (16%)
Colorectal	23 (15%)
Renal	4 (3%)
Other^a^	16 (10%)
Unknown	6 (4%)
Number of brain metastases	
1	108(70%)
2–4	37 (14%)
>4^b^	10 (7%)
Location of brain metastases	
Supratentorial	101 (65%)
Infratentorial	54 (35%)
ECOG performance status	
0	36 (23%)
1	69 (45%)
2	34 (22%)
3	11 (7%)
4	3 (2%)
Not registered	2 (1%)
Extracranial metastases present	88 (57%)
Postoperative radiotherapy^c^	
None	39 (25%)
Stereotactic radiotherapy	77 (50%)
Whole-brain radiotherapy	24 (15%)
Partial brain radiotherapy	11 (7%)
Unknown	4 (3%)
Grade of resection	
Gross total resection with no brain metastases remaining	69 (44%)
Gross total resection with other brain metastases remaining	23 (15%)
Subtotal resection	59 (38%)
Unknown	4 (3%)

Abbreviation: ECOG, Eastern Cooperative Oncology Group.

^a^RT within 100 days with postoperative intention.

^b^Maximum number of brain metastases was 42.

^c^Oesophageal cancer, testicular cancer, ovarian cancer, anal cancer, thyroid cancer, bladder cancer, uterine cancer.

GTR of all present brain metastases was achieved in 69 (44%) patients, while 23 (15%) achieved GTR of 1 brain metastasis but had remaining metastases elsewhere in the brain (GTR with multiplicity). Subtotal resection was performed in 59 (38%) patients, with 4 cases where the extent of resection could not be determined. Postoperative radiotherapy was administered to 112 (75%) patients. Brain metastasis location was identified in 92 of the 93 patients who responded to questionnaires both at baseline and 1 month. We found that 51 patients (55%) had brain metastases in a motor area (the primary motor cortex, supplementary motor area, premotor cortex, corticospinal tract, or in the cerebellum), while 41 (45%) did not.

**Figure 1.**
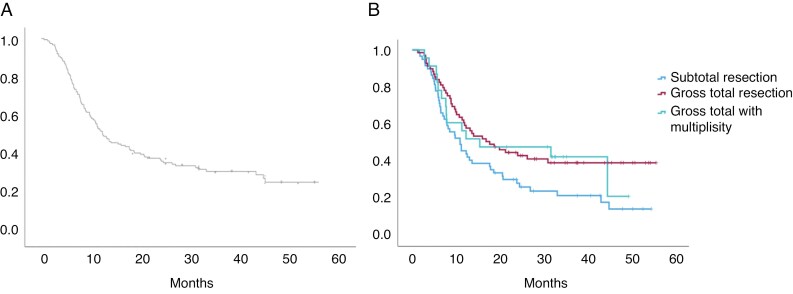
(a) Overall survival after surgery for brain metastases. (b) Overall survival based on extent of resection in patients with brain metastases.

The median OS after surgery for brain metastases was 13 months (95% CI: 8.7–17.5). Patients who underwent GTR had a significantly longer median OS compared to those who underwent subtotal resection (17.7 months vs. 10.9 months, *P* = .044, Figure 1b).

### Questionnaires

Of the 155 patients, 121 (78%) patients completed the first set of questionnaires at inclusion. The response rate gradually declined, with 78 of the 146 patients alive (53%) responding at 3 months, 63/123 (51%) at 6 months, and 35/81 (43%) at 12 months. At inclusion, the mean score for QoL was 61.8 (SD: 24.6) (Table 2). Any degree of motor dysfunction (score > 0 on a scale of 0–100) was reported by 85 (70%) patients and the mean motor dysfunction score at inclusion was 21.8 (SD: 22.8) (Table 3). Among the 81 patients alive at 12 months there were no relevant differences in age, sex, ECOG status, primary cancer, number of brain metastases, or presence of extracranial metastases between those who did and did not complete questionnaires at 12 months.

**Table 2.** Results EORTC QLQ-C15-PAL for All Responders (*n* = 121)

Domains	Inclusion	Month 1	Month 2	Month 3	Month 6	Month 9	Month 12
Patients alive	155	154	151	146	123	98	81
PROM response rate	121 (78%)	103 (67%)	94 (62%)	78 (53%)	63 (51%)	44 (45%)	35 (43%)

	Mean (SD)						
Overall QoL	**61.8 (24.6)**	**63.8 (22.9)**	**62.3 (24.3)**	**62.2 (23.4)**	**62.4 (22.0)**	**68.3 (18.6)**	**69.0 (18.6)**
Physical function	75.4 (24.6)	76.1 (23.5)	78.7 (20.8)	79.1 (22.6)	79.0 (22.9)	82.3 (23.0)	84.0 (15.1)
Emotional function	79.9 (24.3)	81.7 (22.9)	81.7 (22.4)	82.5 (19.9)	83.6 (18.6)	84.5 (20.8)	86.6 (17.3)
Fatigue	36.6 (26.0)	38.6 (22.6)	40.7 (24.6)	38.0 (25.2)	37.3 (23.9)	30.7 (29.4)	27.8 (22.9)
Nausea/vomiting	11.6 (24.2)	10.9 (20.6)	13.3 (21.5)	15.0 (26.7)	11.8 (21.0)	8.3 (17.8)	6.5 (13.4)
Pain	21.8 (25.3)	21.8 (26.0)	23.7 (27.5)	20.7 (24.8)	19.0 (24.5)	15.5 (21.7)	14.4 (19.2)
Dyspnea	19.0 (27.6)	23.6 (28.3)	21.5 (26.3)	22.2 (26.1)	19.6 (21.3)	18.6 (19.7)	19.4 (23.1)
Sleep disturbance	36.1 (33.4)	25.4 (27.9)	25.8 (30.3)	27.3 (31.9)	23.8 (24.3)	29.5 (28.9)	23.1 (28.5)
Appetite loss	17.8 (31.4)	16.5 (25.2)	18.3 (26.2)	22.1 (29.9)	19.0 (30.9)	12.1 (25.0)	11.1 (19.5)
Constipation	15.4 (25.8)	17.2 (24.3)	18.6 (24.8)	15.0 (20.6)	17.5 (23.8)	15.9 (23.3)	17.6 (27.0)

Overall QoL scores shown in bold values.

**Table 3.** Results EORTC QLQ-BN20 for All Responders (*n* = 120)

Domains	Inclusion	Month 1	Month 2	Month 3	Month 6	Month 9	Month 12
Patients alive	155	154	151	146	123	98	81
PROMs responders (response rate)	120 (77%)	102 (66%)	93 (62%)	77 (53%)	62 (50%)	43 (44%)	36 (44%)
	Mean (SD)						
Headaches	21.9 (29.8)	19.7 (24.7)	19.9 (26.5)	23.1 (27.0)	17.2 (27.5)	12.9 (23.0)	15.7 (24.5)
Visual disorder	11.8 (19.0)	9.8 (18.7)	11.7 (19.3)	10.8 (17.4)	9.7 (15.8)	6.9 (11.6)	9.0 (17.0)
Seizures	2.5 (9.8)	1.6 (8.6)	1.4 (6.8)	2.1 (8.2)	4.8 (14.5)	2.3 (8.6)	1.9 (7.7)
Motor dysfunction	**21.8 (22.8)**	**18.8 (21.4)**	**18.8 (18.6)**	**17.9 (21.2)**	**21.5 (24.2)**	**15.2 (18.9)**	**12.3 (15.4)**
Communication deficit	11.8 (18.2)	8.2 (15.6)	9.6 (15.1)	9.1 (14.8)	13.2 (22.9)	9.3 (15.7)	9.6 (14.3)
Drowsiness	28.6 (27.1)	35.3 (24.3)	31.9 (22.4)	33.8 (23.1)	31.7 (24.7)	25.0 (26.0)	22.9 (22.5)
Weakness of legs	17.5 (27.5)	24.5 (28.9)	22.2 (27.1)	23.1 (30.1)	26.5 (30.0)	17.5 (28.7)	13.0 (18.3)

Motor dysfunction scores shown in bold values.

**Figure 2.**
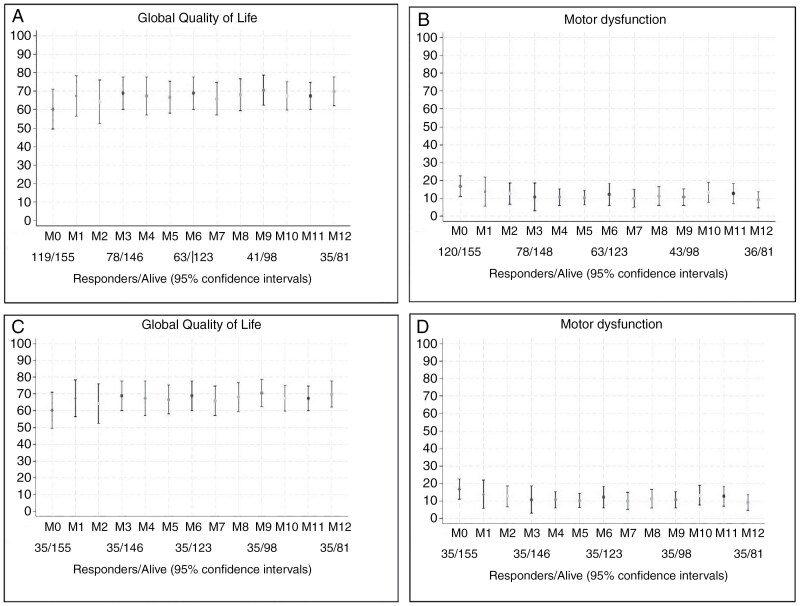
(a) Mean self-reported overall quality of life from inclusion to 12 months for all patients. (b) Mean self-reported motor dysfunction from inclusion to 12 months for all patients. (c) Mean self-reported overall quality of life from inclusion to 12 months for 35 patients who responded at all time points. (d) Mean self-reported motor dysfunction from inclusion to 12 months for 35 patients who responded at all time points.

Overall, QoL scores remained stable (score 61.8–69.0) from inclusion to 12 months (Figure 2a), while mean scores for motor dysfunction improved with 9.5 points (Figure 2b). Analyses conducted on patients with completed questionnaires at all time points showed consistent QoL outcomes (mean score: 61.8–69.0) and a mean change of −3.7 points in motor dysfunction scores from inclusion to 12 months (Figure 2c and d).

To further investigate a potential detrimental impact of surgically induced neurological damage, we looked at the difference in pre- and postoperative self-reported motor dysfunction 1 month after surgery. In total, 120/155 (77%) patients completed the items on motor dysfunction at inclusion. All 120 patients were alive after 1 month with 93 patients responding to the questionnaires. At first follow-up, 35/93 (38%) patients reported no change in motor dysfunction, 28/93 (30%) reported improvement, and 30/93 (32%) reported worsened motor dysfunction. Twenty-seven patients did not respond at 1 month, resulting in a total of 62/155 (40%) patients that did not complete questionnaires both at baseline and 1 month after surgery. Patients who experienced improvement in motor dysfunction 1 month after surgery had a high motor dysfunction score preoperatively (mean 37.5) compared to patients who reported worsened motor dysfunction (mean 15.9), or no difference (mean 8.9). The rate of GTR did not differ between the groups. Brain metastasis location (motor area vs. non-motor area) was not significantly different between patients who reported better, worse, and no change in motor dysfunction (*P* = .98) (Table 4).

**Table 4:** Self-Reported Motor Dysfunction 1 Month After Surgery (Number of Respondents Both at Baseline and 1 Month = 93/155)

Patient Characteristics	Did Not Complete Questionnaires at Inclusion and/or 1 Month *N*: 62/155	No Difference *N*: 35/93 (38%)	Improved Motor Dysfunction *N*: 28/93 (30%)	Worse Motor Dysfunction *N*: 30/93 (32%)
ECOG status				
0–1	37 (60%)	28 (80%)	17 (61%)	23 (77%)
2	17 (27%)	6 (17%)	6 (21%)	5 (17%)
3–4	7 (11%)	1 (3%)	4 (14%)	2 (7%)
Missing	1	0	1	0
Primary cancer				
Lung	19 (31%)	13 (37%)	11 (39%)	9 (30%)
Melanoma	11 (18%)	7 (20%)	5 (18%)	7 (23%)
Breast	12 (19%)	5 (14%)	4 (14%)	4 (13%)
Colorectal	8 (13%)	6 (17%)	5 (18%)	4 (13%)
Other^a^	12 (19%)	4 (11%)	3 (11%)	6 (20%)
Number of brain metastases				
1	48 (77%)	24 (68%)	18 (64%)	18 (60%)
2–4	12 (19%)	9 (26%)	8 (29%)	8 (27%)
>4	2 (3%)	2 (6%)	2 (7%)	4 (13%)
Age ≥70	23 (37%)	16 (46%)	9 (32%)	13 (43%)
Extracranial metastases present	35 (56%)	21 (60%)	15 (54%)	17 (57%)
Brain metastasis location				
Motor area		19 (54%)	12 (43%)	13 (43%)
Non-motor area		16 (46%)	15 (54%)	17 (57%)
Missing		0	1	0
Extent of resection				
Gross total resection	31 (50%)	16 (46%)	12 (43%)	10 (33%)
Gross total resection with other brain metastases remaining	4 (6%)	5 (14%)	4 (14%)	10 (33)
Subtotal resection	24 (39%)	14 (40%)	11 (39%)	10 (33)
Unknown	3 (5%)	0 (0%)	1 (4%)	0 (0%)
Motor dysfunction score at inclusion (mean)	28.8 (SD: 22.3)	8.9 (SD: 13.4)	37.3 (SD: 26.6)	15.9 (SD: 17.4)
Motor dysfunction score at 1 month (mean)		8.9 (SD: 13.4)	15.5 (SD: 20.6)	33.7 (SD: 22.7)
Difference in motor dysfunction score		0.0	−21.8 (SD: 14.7)	17.8 (SD: 11.5)

Abbreviation: ECOG, Eastern Cooperative Oncology Group.

^a^Renal cancer, esophageal cancer, testicular cancer, ovarian cancer, bladder cancer, uterine cancer, and origo incerta.

A multiple linear regression model for motor dysfunction score at 1 month postoperatively identified age above 70 years, high motor dysfunction at inclusion, and multiple brain metastases to be associated with higher motor dysfunction score at 1 month. Brain metastasis location (motor area vs. non-motor area), GTR, status of extracranial disease (stable/progressive), and ECOG performance status at inclusion were not associated with motor dysfunction score at 1 month.

**Figure 3.**
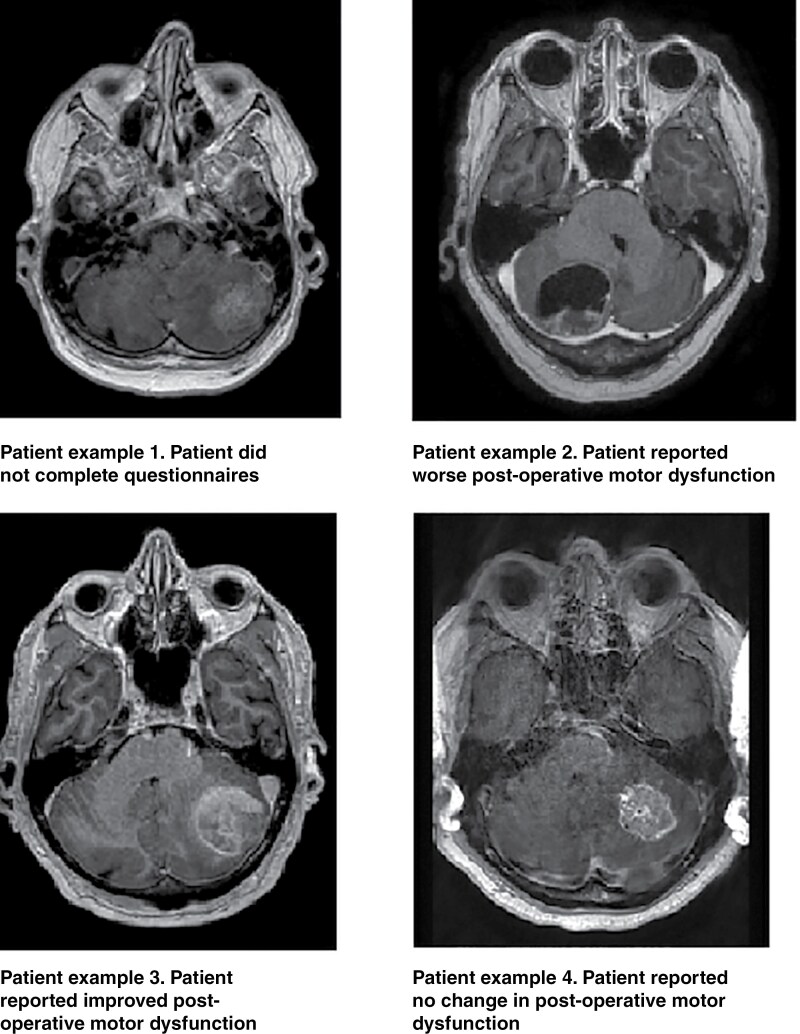
Cerebral MRI examples.

We found no significant difference in OS between patients who reported no change in motor dysfunction at 1 month (median 17.9 months, 95% CI: 9.8–26.0) compared to those who reported improved motor dysfunction (18.6 months, 95% CI: 0.0–45.0), or compared to those reporting a worsening (17.7 months, 95% CI: 4.1–31.3). There was no difference in questionnaire completion rate between the 3 groups at 12 months. Patients who reported worse motor dysfunction at 1 month had higher mean motor dysfunction score at 12 months (21.2) compared to those who reported unchanged or improved scores at 1 month (10.1 and 3.7, respectively). Examples of cerebral MRIs in patients with different motor dysfunction outcomes after surgery are shown in Figure 3.

## Discussion

In this study, we investigated survival, self-reported QoL, symptoms, and motor dysfunction following surgery for brain metastases. Our findings reveal that mean scores for self-reported QoL and symptoms, including motor dysfunction, remained stable or improved post-surgery. The median OS observed in our study was comparable to or longer than that reported in similar studies of patients undergoing surgery for brain metastases.^25–27^

The proportion of patients who completed questionnaires both at inclusion and 12 months was of the same magnitude as in similar studies.^28–30^ Nonresponders constitute a problem in research with patient-reported outcomes, and this is especially true in palliative care, where patients who do not respond are likely to be sicker than those responding to questionnaires, creating a nonresponse bias. Therefore, conducting analyses with multilevel models is not necessarily feasible, since one cannot assume that data are missing at random. To minimize nonresponse bias, future studies should aim to increase questionnaire completion rates. A systematic review identified several factors to achieve this, including patient involvement during study protocol, flexible collection of questionnaires, for instance per mail, in the clinic or electronically, in addition to the education of patients on the importance of patient-reported outcomes, and on how to complete the questionnaires.^31^ Another study highlighted that shorter questionnaires, digital and dynamic questionnaires, and strict study logistics could improve questionnaire response rate.^32^ Notwithstanding, QoL at inclusion was consistent with previous research in this patient population.^28^

Among patients with complete questionnaires at all time points, mean motor dysfunction score remained stable at 12 months compared to the time of inclusion. This observation provides valuable insights into the outcomes following surgery, particularly given the challenging nature of brain metastases. Notably, patients who reported improved motor dysfunction shortly after surgery had high preoperative scores, which is mechanistically reasonable given the alleviation of intracranial pressure. Even if the tumor was not located in a motor area, tumor-induced edema may have influenced motor dysfunction. These findings can inform healthcare providers and patients in the decision-making regarding treatment options, although caution is advised due to the risk of regression to the mean with extreme baseline scores.

Even though we observed a high number of patients (55%) with brain metastasis within motor areas, we found no association between change in motor dysfunction 1 month after surgery and brain metastasis location. The patient-reported outcomes were not related to the anatomical localization of tumor. Overall, our data suggest that carefully performed surgery within motor areas is safe with regard to motor outcomes. Surgery in these areas has benefitted from the recent improvements in surgical mapping and monitoring techniques^33^ The limited number of patients in our study does not allow for further dissection of which patients are more at risk for reduced QoL according to tumor localization.

Contrary to previous findings, our study did not find an association between worse motor dysfunction 1 month post-surgery and shorter OS.^15^ This discrepancy may be attributed to the selection of patients responding to questionnaires in our study. Although GTR was associated with longer OS, the small study population limited our ability to conduct regression analyses including other factors associated with survival, such as age, performance status, status of extracranial disease, and primary tumor. Nonetheless, our findings suggest that prioritizing GTR of brain metastases in motor areas is safe with regard to motor function, when technically possible. Other studies have demonstrated that GTR of brain metastases is likely to impact OS.^14,34^ Nevertheless, the decision to pursue surgical resection of brain metastases should always be considered through a multidisciplinary tumor board, with an evaluation of all treatment options for each individual patient.

Worsening motor function may be a natural part of progressive cancer, since subjective perception of gait is likely influenced by general fatigue, extracranial metastases such as bone metastases, cachexia, and systemic cancer treatments, in addition to brain metastases and brain surgery. In this study, we observed a higher percentage (25%) of patients who reported worse motor dysfunction 1 month after brain metastasis surgery compared to previous retrospective studies.^35,36^ However, these studies were based on electronic patient records, without the use of questionnaires. This emphasizes the importance of acknowledging the patients’ own evaluation of symptoms and well-being, to inform decision-making and tailor individual interventions.

### Strengths and Limitations

Strengths of the study include the prospective design and long follow-up. Other strengths are the homogenous population and detailed information on the extent of resection. Further, the use of questionnaires investigating patient-reported symptoms and quality of life is an important benefit. The low questionnaire response rate after 12 months, however, limits the findings. The study would have benefitted from standardized neurological examinations at inclusion and during follow-up, but unfortunately, this was not feasible.

## Conclusions

Our study, utilizing validated questionnaires, demonstrated that QoL, symptom burden, and motor dysfunction remained stable after surgery for brain metastases. GTR of brain metastases is associated with longer OS and does not increase the risk of worsening patient-reported motor dysfunction, even within motor areas of the brain.

## Data Availability

The data presented in this study are available on request from the corresponding author.
